# The Endothelium as a Driver of Liver Fibrosis and Regeneration

**DOI:** 10.3390/cells9040929

**Published:** 2020-04-10

**Authors:** Erica Lafoz, Maria Ruart, Aina Anton, Anna Oncins, Virginia Hernández-Gea

**Affiliations:** 1 Unidad de Hemodinámica Hepática, Servicio de Hepatología, Hospital Clínic, Universidad de Barcelona, Instituto de Investigaciones Biomédicas Augusto Pi Suñer (IDIBAPS), 08036 Barcelona, Spain; LAFOZ@clinic.cat (E.L.); maria.ruart.millan@gmail.com (M.R.); anton@clinic.cat (A.A.); aoncins@clinic.cat (A.O.); 2 Centro de Investigación Biomédica en Red de Enfermedades Hepáticas y Digestivas (CIBERehd), Instituto de Salud Carlos III, 28029 Madrid, Spain

**Keywords:** liver, liver sinusoidal endothelial cells, LSEC, hepatic stellate cells, endothelial dysfunction, oxidative stress, inflammation, liver fibrosis resolution, liver regeneration, LSEC targeting

## Abstract

Liver fibrosis is a common feature of sustained liver injury and represents a major public health problem worldwide. Fibrosis is an active research field and discoveries in the last years have contributed to the development of new antifibrotic drugs, although none of them have been approved yet. Liver sinusoidal endothelial cells (LSEC) are highly specialized endothelial cells localized at the interface between the blood and other liver cell types. They lack a basement membrane and display open channels (fenestrae), making them exceptionally permeable. LSEC are the first cells affected by any kind of liver injury orchestrating the liver response to damage. LSEC govern the regenerative process initiation, but aberrant LSEC activation in chronic liver injury induces fibrosis. LSEC are also main players in fibrosis resolution. They maintain liver homeostasis and keep hepatic stellate cell and Kupffer cell quiescence. After sustained hepatic injury, they lose their phenotype and protective properties, promoting angiogenesis and vasoconstriction and contributing to inflammation and fibrosis. Therefore, improving LSEC phenotype is a promising strategy to prevent liver injury progression and complications. This review focuses on changes occurring in LSEC after liver injury and their consequences on fibrosis progression, liver regeneration, and resolution. Finally, a synopsis of the available strategies for LSEC-specific targeting is provided.

## 1. Introduction

Liver injury of any kind induces several molecular changes that eventually lead to the progressive fibrosis of the parenchyma and development of liver cirrhosis, the end stage of chronic liver disease. Changes at the liver endothelium level are crucial in the pathogenesis of liver fibrosis and represent the main focus of this review.

The liver has a unique vascular supply where two main venous vascular systems (portal vein and inferior cava vein) and the hepatic artery interact. Vessels ramify successively into more branches and capillaries until converging and forming a vascular network that coats the hepatic sinusoid. Liver sinusoidal endothelial cells (LSEC) are a highly specialized and distinctive micro-vascular cell type, key in the regulation of the liver microenvironment [1,2]. They are the only endothelial cells in the organism lacking a basal membrane and containing small pores called fenestrae. Fenestrae entail open channels that allow bidirectional blood flow between the sinusoidal blood and the hepatic cells [3,4]. LSEC are first defense barrier and contribute to hemostasis/thrombosis, metabolite transport, inflammation, angiogenesis, and vascular tone regulation. Moreover, they participate in the liver cellular response to a given injury by regulating the neighboring cells [1,5,6], mainly hepatic stellate cells (HSC), the principal source of extracellular matrix (ECM) and the key player in fibrosis progression [7]. Indeed, fibrosis is the accumulation of ECM that occurs initially as a reversible wound-healing response, after acute or chronic injury, irrespective of the underlying etiology (viral infection, alcohol and metabolic injury, drug toxicity…) [8,9]. If the harmful stimulus is acute, ECM deposit is an attempt to limit organ damage that can be degraded when the injury is resolved. However, if the damaging stimulus exceeds the regenerative capacity of the liver and the injury persists, the response becomes excessive. Perpetuation of this “curative” response translates to fibrosis progression, substituting hepatic tissue by a fibrous scar disrupting vascular architecture and liver parenchyma [8,10]. Due to their privileged situation and intimate contact with the blood stream, LSEC are the first liver cell type sensing the toxic stimuli. At very initial phases, LSEC change their phenotype: lose their characteristic fenestrae and develop a basal membrane becoming a continuous endothelium, a process called capillarization. The loss of LSEC phenotype has been identified as the initial trigger to the fibrotic response [11,12,13].

LSEC participate in fibrosis through the secretion of angiocrine signals that act as paracrine factors balancing the liver response to injury towards fibrosis or regeneration [14].

Considering the new discoveries highlighting the role of LSEC as a principal regulator of initial response to damage, this review is divided into five sections. The first section gives a brief overview of how liver injury selectively damage LSEC. The second section focuses on the current knowledge of endothelial dysfunction, regarding how LSEC respond to injury, drives initiation and progression of fibrosis, taking into account autocrine and paracrine communication with parenchymal and non-parenchymal cells. The third section summarizes the role of LSEC in liver regeneration. The fourth section examines how LSEC are involved in fibrosis resolution. Finally, in the fifth section LSEC are presented as a potential target for therapy.

## 2. Triggers for LSEC Dysfunction

LSEC have an important role in the early response to liver injury as they orchestrate the initial response to damage of the neighboring hepatic cells. Therefore, a better understanding of the pathways involved in the initiation of the wound healing response and perpetuation of the fibrotic process are crucial to identify relevant therapeutic targets able to modify fibrosis natural history.

A wide range of LSEC noxious stimuli exist, of which ethanol [15], triglycerides and free fatty acids (FFAs) [16], hepatitis C virus (HCV) core protein [17], and HCV non-structural protein 5A (NS5A) [18] are the most common. Such stimuli trigger endothelial cell dysfunction mainly through generation of reactive oxygen species (ROS) and inflammation [19,20,21,22,23,24].

Oxidative stress is a phenomenon caused by an imbalance between the production of free radicals (species with one or more unpaired electrons), reactive metabolites, or ROS and their elimination through antioxidant mechanisms [25,26,27]. Oxidative stress is able to modify the phenotype of many hepatic cell types including hepatocytes, HSC, and inflammatory cells [28], but LSEC are probably the most sensitive liver cell type to oxidative stress [29,30] due to several reasons: first, ROS have been described as key drivers in the initiation of liver injury and LSEC response [30,31,32,33]; second, ROS selectively damage LSEC and alter LSEC phenotype during liver injury [20,27,29]; and third, LSEC are prone to oxidative stress due to a reduction in their enzymatic detoxifying capacity of H_2_O_2_ [34,35,36]. In addition to the classical antioxidant response, LSEC have additional mechanisms, such as autophagy (a degradation process that maintains LSEC homeostasis), able to detoxify oxidative species and necessary for a proper adaptive response to stress. Indeed, recent work from our team reveals that impaired LSEC autophagy causes an improper response to oxidative damage, aggravates their phenotype, and provokes an impaired response to damage in the liver [37].

Oxidative stress can also be responsible for modulating the expression of pro-inflammatory cytokines and chemokines in inflammatory cells [38] initiating a robust inflammatory response [39].

Inflammation is also initiated as a result of liver damage caused by several aetiologies including infections, tissue necrosis and foreign bodies (such as lipids). Inflammatory mediators (basically IL-β and TNF-α) are released by inflammatory cells, damaged epithelial and/or endothelial cells upon injury [24,40,41] that activate LSEC. Activated LSEC upregulate the expression of adhesion molecules such as selectins, vascular cell adhesion molecule-1 (VCAM-1), and intercellular adhesion molecule-1 (ICAM-1) promoting the adherence of monocytes, neutrophils, and lymphocytes. Activated LSEC also induce secretion of several cytokines, chemokines, growth factors, and eicosanoids contributing to the inflammatory response and, therefore, acquiring an inflammatory phenotype [23,24]. In addition, LSEC are able to activate the immune cascade by themselves [42,43,44] by activating the inflammasome [45] as they express pattern recognition receptors such as the mannose receptor, stabilins or TLR4 being able to directly sense danger-associated molecular patterns (DAMPs) or alarmins that typically originate from damaged hepatocytes (like free DNA, mitochondrial DNA, HMGB-1, IL-33, cholesterol, or FFAs) [46,47] and pathogen-associated molecular patterns (PAMPs) derived from microorganisms. Importantly, TLRs when activated are also able to activate LSEC and contribute to the release of cytokines that facilitate the progression of liver disease [23].

Inflammation *per se* is also able to modify LSEC vasodilatory capacity by reducing NO bioavailability and to increase ROS production by altering mitochondria permeability and fitness, altogether contributing to LSEC dysfunction [48].

## 3. Endothelial Dysfunction and Fibrosis Progression

**Figure 1:**
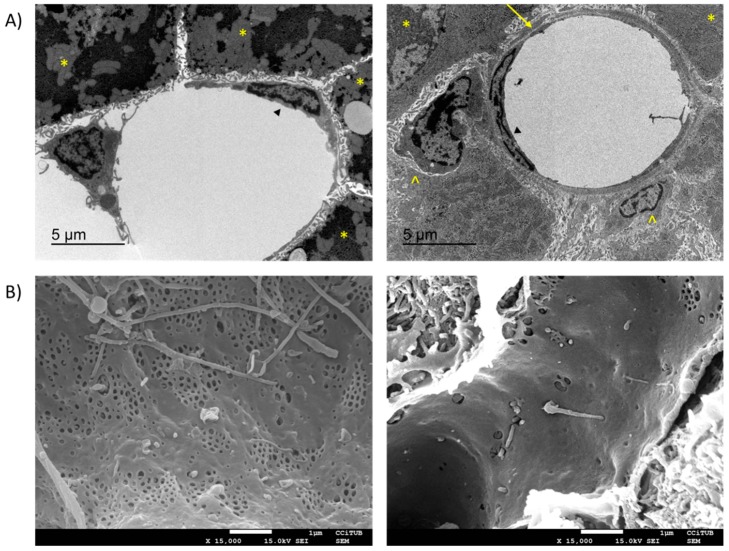
Structural changes in liver sinusoidal endothelial cells (LSEC) after chronic liver injury. (**A**) TEM images from a control liver (left) and a CCl_4_ induced cirrhosis (right). LSEC (►), hepatocytes (*), and HSC (^) are marked. Cirrhotic liver displays a basal membrane (arrow) which is not found in healthy liver. (**B**) SEM images (8000×) of fenestrae in sinusoids of healthy LSEC (left) and LSEC from CCl_4_ induced cirrhosis (right). LSEC from cirrhotic rats show an important loss of fenestrae in comparison with healthy rats. Original images taken by the authors from Wistar control rats (left) and CCl_4_ induced decompensated cirrhosis (right).

In homeostatic conditions, LSEC are more than a fenestrated endothelium; they exhibit a vasodilatory, anti-inflammatory, anti-thrombotic, and anti-fibrotic phenotype [2]. They also regulate angiogenesis and regeneration and are very sensitive to the mechanical forces generated within the microenvironment. After a sustained hepatic injury, LSEC rapidly change their phenotype, become capillarized and acquire a pro-vasoconstrictive, pro-inflammatory, pro-thrombotic, pro-angiogenic and pro-fibrotic phenotype that impair the liver regenerative response in a process called endothelial dysfunction (Figure 1) [49,50,51,52,53,54].

**Figure 2:**
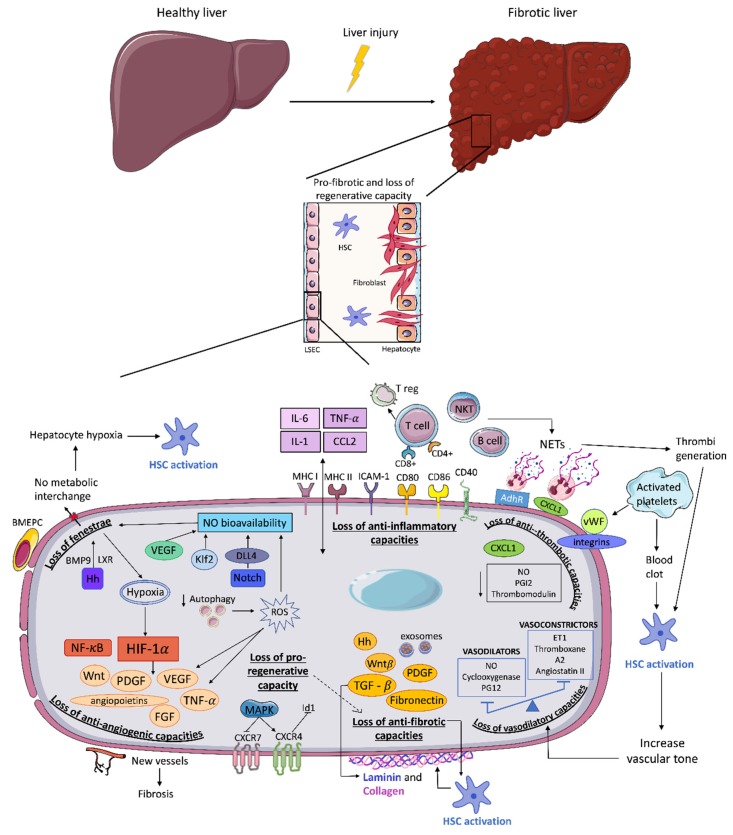
Changes in LSEC associated to endothelial dysfunction. After a liver injury LSEC undergo several changes: the loss of fenestrae and loss of anti-inflammatory, anti-thrombotic, anti-angiogenic, pro-regenerative, anti-fibrotic, and vasodilatory capacities leading to perpetuation of liver fibrosis and impairing liver regeneration. BMEPC: bone marrow endothelial progenitor cells; NETs: neutrophil extracellular traps; ROS: reactive oxygen species.

Recently generated data demonstrate that endothelial dysfunction occurs prior to fibrosis initiation independently of the origin of damage [55,56,57,58,59]. Moreover, DeLeve and co-workers [11] verified that LSEC prevent HSC activation promoting its reversion to quiescence, suggesting that a preserved LSEC phenotype is essential to halt fibrosis progression. Interestingly, phenotypic changes in LSEC appear at early phases in dissimilar liver aetiologies such as non-alcoholic fatty liver disease (NAFLD) and alcoholic liver damage. It has also been described that LSEC dysfunction precedes Kupffer cell (KC) activation, reduction of nitric oxide content, NF-kB activation, and TNFα, IL-6 and ICAM-1 up-regulation [56,58,59,60,61,62]. Therefore, a better understanding of the mechanisms implicated in the loss of LSEC functional capacity and their contribution to the initial response to damage is essential to find strategies able to halt or hamper fibrosis progression (Figure 2).

### 3.1. Loss of LSEC Fenestrae

Loss of LSEC fenestrae (capillarization) is the kickoff event in liver fibrosis. It precedes HSC activation and contributes to hepatic fibrosis and progression [11]. LSEC fenestrae are dynamic structures forming a semipermeable membrane, maintained by a cytoskeleton ring made up of actin and myosin [63,64]. Those fenestrae are usually open and allow the bidirectional metabolic exchange of molecules, lipoproteins, oxygen, small chylomicrons remnants and small particles between the blood and the parenchymal cells. Number and diameter of fenestra can be modulated by several factors, such as blood pressure, hormones, drugs or even changes in the ECM, among others. Substances such as serotonin, α-adrenergic agonists and long-term ethanol abuse lead to a decreased diameter of fenestrae [65]. Narrowing of the fenestrae may impair the pass of molecules, increasing the deposition of triglyceride-rich chylomicron remnants in vascular beds and perpetuating liver injury. Moreover, losing fenestrae may also imply a decrease in the clearance of pharmaceutical agents and less interactions between Kupffer cells and hepatocytes.

Interestingly, defenestration is a dynamic process and it can be reverted upon removal of the trigger [65].

Capillarization is accompanied by the development of a basement membrane; LSEC lose discontinuity and become a continuous endothelium. The basement membrane created by deposition of ECM and interstitial collagen in the space of Disse also contributes to the loss and closure of fenestra [66], impeding the metabolic interchange and aggravating hepatocyte hypoxia, a potent trigger of HSC activation and fibrogenesis [54].

The exact mechanisms regulating the loss of fenestra have not been fully elucidated but several pathways have been identified. Probably the better-known mechanism controlling the LSEC phenotype is nitric oxide (NO). Indeed, several molecules and processes are able to regulate LSEC phenotype by directly controlling NO synthesis and bioavailability. Vascular endothelial growth factor (VEGF) secreted by both HSC and hepatocytes is believed to maintain LSEC phenotype via NO-dependent and NO-independent pathways [12,67,68]. Similarly, Krüppel-like transcription factor, Klf2, maintains a correct phenotype of LSEC up-regulating NO bioavailability [69,70,71]. Endothelial autophagy is also able to control NO bioavailability by regulating the antioxidant response and therefore LSEC capillarization [37]. Notch signaling is also able to promote LSEC dedifferentiation by regulating eNOS/sGC [72] and Delta-like ligand 4 (DLL4) overexpression [73].

Besides NO, Hedgehog (Hh) signaling has also been implicated in LSEC capillarization by regulating liver X receptor (LXR) and BMP9 among others [74,75,76,77,78,79]. Although previously suggested, studies using caveolin-1 knockout mice have demonstrated that caveolin-1 has been observed in fenestrae but it is not involved in capillarization [80,81]. CD47-binding peptide of thrombospondin-1 has also been proposed as a regulator of LSEC defenestration [82].

A recent study has suggested that after a liver injury, bone marrow endothelial progenitors arrive to the site of injury and repopulate the sinusoid. However, their immature nature made them unable to develop fenestrae and maintain HSC quiescent [83].

### 3.2. Loss of Vasodilatory Capacities

One of the key events associated with endothelial dysfunction is the deregulation of the vascular tone. During liver injury, LSEC reduce their capacity to produce and respond to vasodilators, mainly NO, cyclooxygenase, and prostaglandin I2 (PGI2) [84,85] and increase the production of vasoconstrictors (Endothelin1, thromboxane A2, angiotensin II) [85,86]. This disequilibrium does not only alter LSEC phenotype but also contributes to HSC activation [31,74,75,86,87,88,89,90,91] perpetuating its activation and contractibility [31,84,85,86,92]. Moreover, activated HSC have contractile capacity and, as they lie above the endothelium, they further increase the vascular tone [86,92].

### 3.3. Loss of Anti-Inflammatory Capacities

LSEC are an integral part of the hepatic reticuloendothelial system that have a unique immunological role due to their privileged localization in intimate contact with the splanchnic blood. Under physiological conditions, the liver has a unique potential to modulate immune response, especially through tolerance induction. Its continuous exposure to bacterial-derived products from the gut induces the expression of anti-inflammatory cytokines to maintain the state of immune unresponsiveness [42,43,44,45]. However, in presence of damaging stimuli, a robust immune response can be generated and LSEC become highly proinflammatory and start secreting a vast array of cytokines and chemokines (TNF-α, IL-6, IL-1 and CCL2) [93,94,95] capable of activating KC. Moreover injured hepatocytes and inflammatory cells release inflammatory mediators able to further activate LSEC and perpetuate the inflammatory response [93,94,96].

Activated LSEC upregulates the cellular adhesion molecules ICAM-1, VCAM-1, and VAP-1, which recruit blood leucocytes, losing their physiological barrier capacity and leading to entry of circulating leucocytes within the liver parenchyma. LSEC are then transformed from mediators of tolerance to potent stimulators of immunity and become a critical component of intrahepatic inflammation [93,97,98,99,100,101,102,103,104,105,106,107].

LSEC also express MHC class I, MHC class II, ICAM-1, VCAM-1, and the costimulatory molecules CD80, CD86, and CD40 as professional antigen presenting cells (APCs) [108,109] and play an important role in adaptive immunity. Antigen presentation from LSEC to CD4+ T cells promote the development of regulatory T cells (Tregs) [110]. Tregs stimulate fibrogenesis by inducing Th17 cells activation and increasing the expression of CD8+ and CD4+ T cells [111,112]. 

In a physiological state, antigen presentation from LSEC to CD8 T cells mediates tolerance of naïve CD8(+) T cells [108,113,114,115,116,117], but this response is abrogated when LSEC are exposed to high levels of antigen. During liver injury, naïve CD8 T cells are able to differentiate into effector cells [116] and to induce HSC activation and fibrogenic stimulation [118].

In addition, activated LSEC contribute to the profibrogenic response by recruiting B cells [119], natural killer T cell (NKT) cells [120] and activating KC [120,121,122]. Activated KC secrete a wide range of inflammatory cytokines able to sustain the inflammatory state and activate HSC. As a matter of example, TNF and IL-1β secretion by KC perpetuate hepatocyte injury [123] and CCL2 stimulates the infiltration of CCR2+ Ly-6Chi circulating monocytes in the liver [124,125] capable of activating and promoting HSC proliferation through the secretion of TGFβ1 and PDGF [124]. Therefore, LSEC act as effector cells promoting inflammation and are main targets of inflammatory cells contributing in both ways to amplify the fibrotic response.

### 3.4. Loss of Anti-Thrombotic Capacities

Healthy endothelial cells express molecules that prevent platelet activation, coagulation and thrombus formation [126,127,128,129,130]. During endothelial dysfunction, LSEC lose their antithrombotic phenotype altering the expression of pro- and anti-thrombotic factors [2,130,131]. Dysfunctional LSEC expose von Willebrand factor, integrins and other receptors that interact with activated platelets, ultimately leading to blood clot formation [132,133] as well as attenuating the expression of thrombomodulin, NO or PGI2 [132]. LSEC by promoting activation of the coagulation cascade and specially thrombin generation and protease–activated receptors (PARs) can induce microthrombosis and parenchymal extinction, processes linked to fibrosis progression [134,135,136,137,138,139,140].

Dysfunctional LSEC can also contribute to thrombosis through the recruitment of inflammatory cells. Indeed, a recent study by Hilscher et al. [141] has directly implicated LSEC in the formation of sinusoidal microthrombi through the secretion of CXCL1, which mediates neutrophil recruitment and release of neutrophil extracellular traps (NETs) in congestive hepatopathy. Additional studies are needed to evaluate whether this finding translates to other aetiologies.

### 3.5. Loss of Anti-Angiogenic Capacities

Sinusoidal capillarization together with chronic inflammation promote angiogenesis. The loss of fenestrae entails disruption of the oxygen supply and appearance of hypoxia leading to accumulation of hypoxia-inducible transcription factors (HIF) that stimulate the production of angiogenic growth factors (VEGF, FGF, angiopoetins and PDGF among others) by the surrounding cells and start new vessel formation [142,143]. 

Mainly in response to oxidative stress [142,144], LSEC themselves can also promote angiogenesis by directly secreting VEGF [145], TNF-α [142], angiopoietin 2 [146] and various types of Wnt ligands and their frizzled receptors [147].

It has been recently suggested that release of microparticles by liver cells (hepatocytes [148,149], portal myofibroblasts [150], endothelial progenitor cells [151,152], etc.) may induce angiogenesis [153]. Although the direct effects of angiogenesis on fibrosis have not been clarified yet, both processes are closely related [154]. Stimulation of angiogenesis (by deletion of the angiogenesis inhibitor prolyl-hydroxylase-2) results in liver fibrosis accumulation [155]. On the other hand, blocking angiogenesis can aggravate liver fibrosis [156] but also promote fibrosis resolution [157,158] highlighting the importance of the fibrotic stage in the angiogenic response. The exact contribution of angiogenesis to fibrosis deserves further investigation. 

### 3.6. Loss of Anti-Fibrotic Capacities

After a hepatic injury, LSEC themselves acquire a pro-fibrotic phenotype and participate in fibrosis by directly secreting ECM, and indirectly, regulating the hepatic microenvironment via secretion of pro-fibrotic molecules. Their direct contribution is due to the synthesis of laminin and collagen in response to TGFβ1 after sustained injury [159]. Whether endothelial cells generated from endothelial-to-mesenchymal transition [160] may contribute to ECM deposition remains a field of research.

LSEC contribute to fibrosis mainly through HSC activation. As mentioned before, LSEC regulate HSC activation via an alteration of the balance of vasodilators/vasoconstrictors molecules. LSEC also contribute to HSC activation through additional ways: secreting fibronectin EIIIA [161,162], TGF-β and PDGF or activating signaling pathways such as Hh or Wntβcatenin, which can activate HSC in both a paracrine and an autocrine manner [72,74,75,76,163,164,165,166,167,168,169,170].

Exosomes have been proposed as a novel way of intercellular communication through protein and lipid exchange [171]. The role of exosome signaling in HSC-LSEC crosstalk promoting LSEC dysfunction during liver fibrogenesis [75] has been described to be bidirectional. It has been shown that exosomes derived from dysfunctional LSEC (containing sphingosine kinase 1, SK1) regulate HSC activation and migration favoring fibrogenesis [172]. Further studies are needed to really understand the contribution of exosomes and their cargo to the fibrotic process. 

### 3.7. Loss of Pro-Regenerative Capacity

Healthy liver has the ability to regenerate after injury, but when the regenerative response is insufficient or exceeded, fibrosis develops [173,174]. The groundbreaking work of Ding et al. highlights the crucial role of LSEC in the liver regenerative response [175] by controlling hepatocyte proliferation. LSEC orchestrate the response of the liver microenvironment and balance regeneration over fibrosis. However, aberrant activation of LSEC in the context of chronic injury provokes the loss of their regenerative capacity and causes liver fibrosis [173,174].

## 4. Effect of Mechanical Forces on LSEC Phenotype

Mechanical forces have also arisen in the recent years as key regulators of LSEC function and phenotype.

### 4.1. ECM Stiffness

Fibrosis entails the accumulation of ECM within the liver parenchyma and increases its stiffness [176]. ECM stiffness modulates cellular behavior [177,178,179,180,181] and studies in vitro have demonstrated that it contributes to LSEC dysfunction by changing LSEC phenotype [182], inducing pseudocapillarization and increasing actin stress fiber formation [96,182,183] and expression of adhesion molecules [182]. Therefore, increased matrix stiffness generated by HSC activation and ECM deposition can further aggravate LSEC dysfunction, perpetuating fibrosis progression [184,185,186,187].

### 4.2. Shear Stress

Physiological shear stress, the frictional force applied by blood flow on the endothelial surface [188], is also a known regulator of endothelial cell behavior and phenotype [84,189,190,191,192], in part through Klf2 expression [193,194,195]. Liver fibrosis is accompanied by several alterations in the vasculature, especially when portal hypertension appears (increased intrahepatic resistance, augmented portal vein flow, angiogenesis, portosystemic collaterals…). Although some evidences suggest that shear stress acting on LSEC following resection could be a key factor in regulating liver regeneration [196,197], the real contribution of shear stress to LSEC dysfunction and its potential therapeutic targeting remain to be elucidated. 

## 5. Role of LSEC Balancing Liver Regeneration and Fibrosis

Liver regeneration involves a synchronized cooperation between parenchymal and non-parenchymal cells [198,199,200,201,202,203] where LSEC orchestrate the secretion of cytokines and growth factors necessaries for hepatocyte proliferation. At the same time, LSEC proliferation is also regulated by hepatocytes and other non-parenchymal cells [14,165]. During regeneration after partial hepatectomy, there are two clearly different phases; a first early/inductive phase of hepatocyte proliferation (first 48 h) followed by a second phase of endothelial cell proliferation (between 72–96 h). It has been demonstrated that LSEC have a role in both phases. LSEC control the secretion of angiocrine factors such as hepatocyte growth factor (HGF), Wnt2, angiopoietin-2, fibronectin Extra Domain A and activin A [165,175,204,205,206,207,208,209,210,211,212,213,214] during the first inductive phase, but their response to hepatocyte-derived VEGF/angiopoietin stimulation [213,215] is also essential for the proliferative phase.

**Figure 3:**
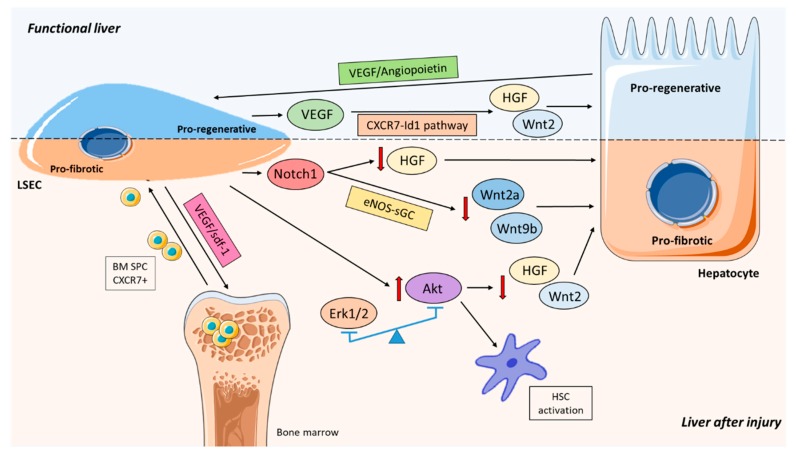
Main signaling pathways involving LSEC during liver regeneration. Crosstalk between hepatocytes and LSEC is crucial during regeneration. In a healthy liver, VEGF pathways induce a pro-regenerative state whereas after liver injury Notch and Akt pathways are activated leading to a pro-fibrotic phenotype. In addition, bone marrow cells (BM SPC) may be recruited by LSEC through VEGF/sdf-1 pathway and engraft in the liver.

Sustained liver damage provokes aberrant LSEC activation and dysfunction compromising their regenerative capacity and shifting the liver response towards fibrosis [173,174] (Figure 3).

The master regulator of the regenerative response seems to be VEGF as it is able to promote hepatocyte and LSEC proliferation [213,216]. VEGF stimulates the release of HGF from LSEC through VEGFR1 promoting hepatocyte proliferation [209]. Moreover, VEGF/VEGFR2 drives regeneration depending on the Id1 pathway. Liver injury activates the CXCR7-Id1 pathway in LSEC in a VEGF dependent manner [165]. In addition, blocking Id1 impairs HGF and Wnt2 secretion by LSEC and regeneration is abrogated. Transplantation of LSEC from WT into Id1-KO mice restores their regeneration capacity after partial hepatectomy, further supporting the central role of the Id1 pathway [165]. Also in the clinical scenario of acute-on-chronic liver failure, it has been demonstrated that defects in the CXCR7-Id1 dependent HGF expression by LSEC impairs regeneration [217].

A recent paper has revealed the role of endothelial Notch in reshaping the angiocrine functions of LSEC leading to more liver fibrosis and impairing liver regeneration in mice [72]. They demonstrated that Notch activation downregulates critical hepatocyte mitogens (Wnt2a, Wnt9b, and HGF) and compromise hepatocyte proliferation. They showed that Wnt2a and Wnt9b act on an eNOS-sGC-dependent manner, but not HGF.

The availability of the Erk1/2-Akt pathway to balance the pro-regenerative or pro-fibrotic phenotype of LSEC has also been suggested. Erk1/2 expression was associated with preservation of a pro-regenerative phenotype of LSEC and HSC quiescence through NO regulation and controlling the antioxidant response, but also via increase of the mitogens HGF and Wnt2. Indeed, Akt upregulation provoked LSEC pro-fibrotic phenotype promoting HSC activation and reducing NO, HGF, and Wnt2 expression [218].

There are several pieces of evidence that support LSEC as drivers of liver regeneration [165,207,209]. Apart from their paracrine functions, LSEC also participate in tissue repair by controlling the generation of new vessels. This can occur due to the extension of resident endothelial cells [219], but also due to the recruitment of bone marrow sinusoidal progenitor cells (BM SPC) that differentiate to LSEC in the site of injury, promoting neovascularization, and becoming a source of HGF [210,211,220,221,222,223,224,225,226]. It has been recently shown that, after several forms of liver injury or partial hepatectomy, lost or injured LSEC are not replaced by mature LSEC during regeneration but by BM SPC or sprocs [206,210,211,227,228]. Indeed, during liver regeneration, VEGF-sdf1 signaling induces proliferation in the bone marrow, mobilization to the circulation, recruitment and engraftment of CXCR7+ (sdf1 chemokine receptor or CXCL12) BM SPC. BM SPC engraft into the liver and differentiate into fenestrated LSEC through NO pathway [206,210,211].

However, to date there is considerable concern about whether BM SPC can physically incorporate into the regenerative vasculature or if they stimulate liver regeneration through secretion of paracrine factors [229,230,231]. In this regard, Singha and co-workers have nicely shown that depending on endothelial cell fitness, the source of regenerating liver vasculature may be different; when the endothelium is intact, neoangiogenesis is only mediated by proliferation of resident endothelial cells but after endothelial cell damage induced by irradiation, BM SPC are recruited and incorporated into the vasculature. They use multiple irradiation-based myeloablative and non-myeloablative mouse models to analyze the contribution of different cellular sources to liver regeneration after partial hepatectomy or chronic CCl_4_ induced liver damage and observe that BM SPC do not integrate into liver vasculature if there is no vascular damage. They therefore suggest that in patients with intact liver endothelial cells, BM cellular therapies will not suffice to liver regeneration [232].

There is a lot of evidence supporting the role of LSEC in coordinating the regenerative process. However, after sustained liver injury, aberrant activation of LSEC balance the liver response towards fibrosis over regeneration. LSEC dysfunction encompasses secretion of angiocrine factors that promotes fibrosis and impairs regeneration. However, the role of LSEC during neovascularization/proliferation in liver regeneration is still controversial and needs further investigation. 

## 6. Fibrosis Resolution

Although fibrosis has long been considered an irreversible condition, this paradigm has changed and nowadays it is considered a dynamic and reversible process, even during advanced stages [8,233,234,235,236,237,238,239]. Fibrosis resolution/regression is the process that takes place after removal of the etiological agent and courses with ECM degradation and hepatocyte regeneration leading to normal or nearly normal liver histology and function [240,241,242,243]. ECM degradation is mainly directed by the balance of matrix metalloproteases (MMPs) and tissue inhibitors of MMPs (TIMPs) produced by a variety of cell types in the liver [242,243,244,245].

LSEC in particular produce VEGF [145], a key mediator in the resolution process as it facilitates liver sinusoidal permeability and recruits monocytes [157] mainly through the CXCL9–MMP13 axis. Recruitment of the monocyte-macrophage lineage, by controlling the secretion of different MMPs and TIMPs [246,247] plays a crucial role in the resolution process [248].

VEGF increases significantly during fibrosis resolution, produced by LSEC and myeloid cells [158]. VEGF fosters a LSEC pro-resolution phenotype with increased expression of MMP-2 and MMP-4, reduced expression of TIMP-1 and TIMP-2 and increased MMP-13 expression in macrophages [158,249], altogether promoting hepatic scar vascularization and fibrosis resolution. Importantly, fibrolytic properties have been attributed to LSEC that following VEGF overexpression are able to accelerate matrix degradation and improve liver regeneration [249]. Therefore VEGF may have a dual role in fibrosis; it is essential in maintaining LSEC physiological phenotype through NO regulation [250] during fibrosis onset and promote reversion of HSC activated phenotype, ameliorating LSEC dysfunction during fibrosis resolution [12].

LSEC may also contribute to the resolution process by other means. They coordinate the recruitment of many other immune cells as previously discussed such as neutrophils, directly involved in MMP secretion and matrix degradation [248]. They can also endocytose and clear denatured collagen α chains from blood due to their mannose receptors [251,252,253,254,255].

Interestingly, a recent study has shown that LSEC express AKAP12, a scaffold protein that integrates several effector proteins, after the withdrawal of fibrotic damage. This protein seems to be important during fibrosis resolution as well, as knocking it out leads to reduced fibrosis resolution [256], although the exact mechanism remains to be investigated.

## 7. LSEC Targeting: Potential for Therapy

LSEC are potent liver scavengers, able to endocytose soluble macromolecules and small particles through their numerous receptors [32,87], making them suitable for specific drug delivery. For this reason, several strategies for specific LSEC-targeting have been developed involving the use of nanoparticles, liposomes, nanoassociates, nanogels, nanocapsules or quantum dots specifically decorated, with the final aim of being directed to LSEC through the recognition of their different receptors.

Hyaluronic acid (HA) can be uptaken by LSEC via different receptors such as CD44, receptor for hyaluronate-mediated motility (RHAMM) and the HA receptor for endocytosis (HARE) or stabilin-2 [257,258]. KC are also able to uptake HA but in a much lower proportion [259]. As a result, this ligand has been explored to increase LSEC delivery by decorating cationic liposomes [259], DNA nanoassociates [260], nanocapsules [261,262] and nanogels [263]. Chondroitin sulphate is also a ligand of the scavenger receptor stabilin-2. Its use as a nanoparticle coating also effectively targets LSEC [264]. Serum albumin targets almost entirely stabilin-2 receptors [265]. This ligand has been used to increase LSEC delivery by coating liposomes [266], lipid particles [267] and quantum dots [268]. Apolipoprotein B, or a part of its sequence, that is also a ligand for scavenger receptors stabilin-1 and 2 has been used to decorate nanoparticles [269,270] and liposomes [271]. Furthermore, the polymer poly(maleic anhydride-alt-1-octadecene) has also been used to coat superparamagnetic iron oxide nanoparticles in order to direct them to the sinusoidal endothelium [272]. Besides that, adenovirus with the endothelial cell specific ROBO4 promoter have been seen to present a high degree of LSEC specificity and have been used to promote liver regeneration while bypassing fibrosis [218]. Finally, quantum dots (QDs), a type of nanoparticles with a size between 1–20 nm that present quantum effects [265] are also an area or interest [265]. Telluride/cadmium sulphide QDs [273] and Zn-labelled CdSelenide/CdS/ZnS QDs have been shown to effectively ameliorate LSEC delivery [274]. However, QDs may produce toxicity as they usually contain cadmium or other toxic elements [274].

Besides nanoparticle coating, particle size has to be taken into account since nanoparticles bigger than 250 nm are directly taken up by KC for being too big to pass through fenestrae. Contrarily, those with a diameter between 5–20nm particularly target LSEC [265,275].

For the moment, LSEC targeting strategies have been employed in the context of cancer, autoimmunity, acute liver damage, ischemia-reperfusion injury and haemophilia [5,262,264,270,272,276], but they represent an attractive treatment also for liver fibrosis. 

In fact, LSEC targeted delivery of drugs would represent an exceptional approach to specifically treat dysfunctional LSEC by selectively modifying their dysregulated pathways. Then, strategies that reverse endothelial dysfunction by recovering fenestrae through Notch [73] or Hh inhibition [76], anti-inflammatory capacity through vascular adhesion protein-1 (VAP-1) blockade [100] or TLR-4 inhibition [277], anti-thrombotic capacity through E-selectin/ P-selectin inhibition [133] or recombinant ADAMTS13 [278,279] delivery or anti-angiogenic capacity through angiopoietin-2 inhibition [105] or VEGF-A [280] neutralization, among others, could be specifically directed to LSEC, permitting an increased specificity and bioavailability in the cell of interest together with a reduction of the necessary dose and side effects in other organs/cells.

## 8. Conclusions

In this review, we have summarized the current knowledge about the role of LSEC as the main regulator of initial response to liver damage and their role in sinusoidal communication. The liver endothelium has a unique phenotype responsible for many of its functions. LSEC are gatekeepers of liver homeostasis because of their vasodilatory, anti-inflammatory, anti-thrombotic, anti-angiogenic, anti-fibrotic and pro-regenerative properties. Once liver injury appears, independently of the aetiology, LSEC are the first cells to sense and respond to it due to their privileged situation. A wide range of noxious stimuli can trigger LSEC loss of phenotype, mainly through the generation of ROS and inflammation. Consequently, LSEC acquire a capillarized phenotype, become pro-vasoconstrictive, pro-inflammatory, pro-thrombotic, pro-angiogenic and pro-fibrotic, and impair their liver regenerative response. Each of those phenomena has their specific contribution, promoting fibrosis progression highlighting the core contribution of LSEC in the fibrotic process. 

In addition, LSEC orchestrate the liver regeneration response controlling the hepatic microenvironment by secreting paracrine factors. Aberrant activation of LSEC in the context of chronic injury promotes the loss of their regenerative capacity and balances liver response towards fibrosis over regeneration. Besides their important role in fibrosis progression, LSEC are also implicated in its resolution mainly through the secretion of VEGF and MMPs and by mediating monocyte infiltration in the liver. For all the previously stated reasons, LSEC targeting may be of enormous utility in liver fibrosis treatment. Moreover, LSEC are excellent targets for drug delivery due to their unique properties and there are many strategies able to reach LSEC and modify dysregulated pathways. Selective LSEC targeting appears as an attractive strategy to treat liver fibrosis.

## Abbreviations

APCs	Antigen presenting cells
BMEPC	Bone marrow endothelial progenitor cells
BM SPC	Bone marrow sinusoidal progenitor cells
CD44	Cluster determinant 44
DAMPs	Danger-associated molecular patterns
DLL4	Delta-like ligand 4
ED	Endothelial dysfunction
ET-1	Endothelin-1
ECM	Extracellular matrix
FFAs	Free fatty acids
Hh	Hedgehog
HSC	Hepatic stellate cells
HCV	Hepatitis C virus
HGF	Hepatocyte growth factor
RHAMM	Hyaluronate-mediated motility
HA	Hyaluronic acid
HARE	Hyaluronic acid receptor for endocytosis
HIF	Hypoxia-inducible transcription factors
ICAM-1	Intercellular adhesion molecule-1
Klf2	Krüppel-like transcription factor 2
KC	Kupffer cells
LSEC	Liver sinusoidal endothelial cells
LXR	Liver X receptor
MMPs	Matrix metalloproteases
NETs	Neutrophil extracellular traps
NO	Nitric oxide
NAFLD	Non-alcoholic fatty liver disease
NS5A	Non-structural protein 5A
PAMPs	Pathogen-associated molecular patterns
PGI2	Prostaglandin I2
PARs	Protease-activated receptors
QDs	Quantum dots
ROS	Reactive oxygen species
Tregs	Regulatory T cells
SEM	Scanning electron microscopy
SK1	Sphingosine kinase 1
TIMPs	Tissue inhibitors of matrix metalloproteases
TEM	Transmission electron microscopy
VAP-1	Vascular adhesion protein-1
VCAM-1	Vascular cell adhesion molecule-1
VEGF	Vascular endothelial growth factor
VEGFR-2	Vascular endothelial growth factor receptor-2
